# Oxidative Stress Mediated-Alterations of the MicroRNA Expression Profile in Mouse Hippocampal Neurons

**DOI:** 10.3390/ijms131216945

**Published:** 2012-12-11

**Authors:** Shunjiang Xu, Rui Zhang, Jingya Niu, Dongsheng Cui, Bing Xie, Binggui Zhang, Kang Lu, Wenjun Yu, Xueyi Wang, Qingfu Zhang

**Affiliations:** 1 Central Laboratory, The First Hospital of Hebei Medical University, Shijiazhuang 050031, China; E-Mails: sjxu66@hotmail.com (S.X.); doudouzr511@yahoo.cn (R.Z.); dongshengcui@sina.com (D.C.); xb0916@yahoo.cn (B.X.); lukang098@163.com (K.L.); yuwenjun_474885@126.com (W.Y.); 2 Department of General Surgery, Hebei Provincial Geriatric Hospital, Shijiazhuang 050031, China; E-Mails: jyniu163@163.com (J.N.); binggui7183@163.com (B.Z.); 3 Institute of Mental Health, Hebei Medical University, Shijiazhuang 050031, China; E-Mail: ydyywxy@163.com; 4 Department of Burns and Plastic Surgery, The First Hospital of Hebei Medical University, Shijiazhuang 050031, China

**Keywords:** oxidative stress, microRNA, hippocampal neurons, array analysis, Alzheimer disease

## Abstract

Oxidative stress plays a critical role in the etiology and pathogenesis of neurodegenerative disorders, and the molecular mechanisms that control the neuron response to ROS have been extensively studied. However, the oxidative stress-effect on miRNA expression in hippocampal neurons has not been investigated, and little is known on the effect of ROS-modulated miRNAs on cell function. In this study, H_2_O_2_ was used to stimulate the mouse primary hippocampal neurons to develop an oxidative stress cell model. The alterations of miRNAs expression were detected by microarray analysis and five miRNAs were validated by real-time RT-PCR. The bioinformatic analysis of deregulated miRNAs was performed to determine their potential roles in the pathogenesis of neurological disorders. We found that H_2_O_2_ mediated a total of 101 deregulated miRNAs, which mainly took part in the regulation of the MAPK pathway. Among them, miR-135b and miR-708 were up-regulated significantly and their targets were predicted to be involved in DNA recombination, protein ubiquitination, protein autophosphorylation and development of neurons. These results demonstrated that oxidative stress alters the miRNA expression profile of hippocampal neurons, and the deregulated miRNAs might play a potential role in the pathogenesis of neurodegenerative diseases, such as Alzheimer’s disease (AD).

## 1. Introduction

Reactive oxygen species (ROS) are chemically reactive molecules containing oxygen. ROS are produced in all aerobic cells. If the generation of ROS in a system exceeds its ability to eliminate them, oxidative stress will occur. Excessive production of ROS will disrupt the cellular redox balance and trigger the oxidation of biological macromolecules, including proteins, lipids and DNA, and as a result, it potentially leads to biological function disorder [1]. In humans, because the brain is responsible for about 20% of the body oxygen consumption, it is easily subjected to an oxidative damage of ROS [2,3]. Growing evidence suggests that oxidative stress plays an important role in the etiology and pathogenesis of neurodegenerative disorders, especially in Alzheimer’s disease (AD).

AD, the most prevalent neurodegenerative disorder, with a decline of memory and cognitive functions in a broad range, is a major public health issue all over the world [4]. It is characterized by the reduction of neurons, intercellular neurofibrillary tangles associated with aberrantly hyperphosphorylated tau, extracellular senile plaques composed mainly of Aβ peptides and loss of synaptic function [5]. Although the exact causes of AD are not clear, a complex etiology is likely, involving multiple environmental, age-related, genetic, epigenetic, viral and inflammatory factors [6]. Less than 5% of all AD cases are caused by gene mutation [7]. The mechanisms underlying the pathogenesis of memory impairment in AD are oxidative stress theory, gene mutations theory, β-amyloid cascade theory, Tau protein hypothesis, mitochondrial dysfunction and autophagy [8]. Multiple lines of evidence suggest that progressive oxidative damage is an early event involved in the pathological process of AD [3,9,10]. In addition to impaired oxidative metabolism detected in AD fibroblasts [11,12], the lipid peroxidation, protein oxidation, as well as other oxidative damage markers were all found in AD brains [13].

MicroRNAs (miRNAs) are single-stranded non-coding small RNAs, approximately 22 nucleotides (nt) in length. It is reported that miRNAs play key roles in regulating protein expression by binding completely or partially to target protein-coding mRNAs at the posttranscriptional level for degradation or translational inhibition [14,15]. In the past few years, a number of studies of miRNAs in AD have emerged to support that deregulated miRNAs, such as miR-18b, miR-34c, miR-615, miR-211, miR-216 and miR-325, play important roles in the development and prognosis of AD [16].

Despite the key roles of oxidative stress and deregulated miRNAs in the pathological process of AD, whether deregulated miRNAs are mediated by oxidative stress in hippocampal neurons remains elusive. Hydrogen peroxide (H_2_O_2_) is a stable member of freely diffusible reactive oxygen species. The oxidative activity of H_2_O_2_ may contribute to the loss of synaptic function characteristic of AD through modifications of protein, lipids and DNA [17]. In the present study, H_2_O_2_ was used to stimulate the mouse primary hippocampal neurons as an oxidative stress cell model, and the alterations of miRNAs expression profile were also detected by microarray analysis. The bioinformatic analysis of deregulated miRNAs was also performed to determine whether these miRNAs play a potential role in the pathogenesis of Alzheimer’s disease.

## 2. Results

### 2.1. H_2_O_2_-Induced Cell Viability Loss in Primary Hippocampal Neurons

**Figure 1:**
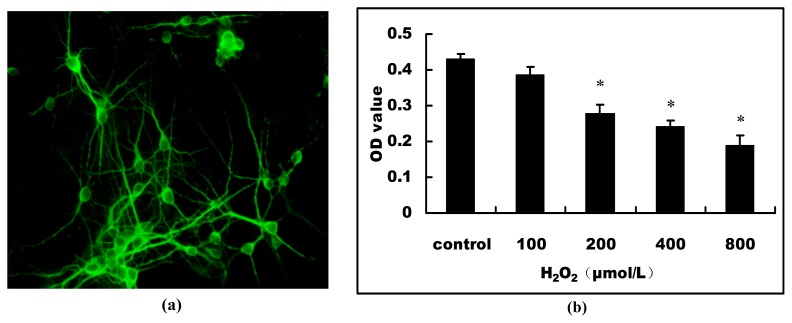
The effect of H_2_O_2_ on the cell viability of mouse primary hippocampal neurons. (**a**) Primary hippocampal neurons grown for 7 DIV were immunostained for MAP-2; (**b**) Cell viability was assessed at 24 h after stimulation with different concentrations (0, 100, 200, 400 and 800 μmol/L) of H_2_O_2_ by MTT assay. * *p* < 0.05 *versus* control.

To illuminate whether neuronal miRNAs are changed by oxidative stress, we stimulated the cultured primary hippocampal neurons with H_2_O_2_ to develop a cell model of oxidative stress. At 7 DIV (days *in vitro*), neurons were matured *in vitro* and had developed dendritic networks shown by neuronal tubulin associated protein (MAP-2) staining (Figure 1a). It is indicated that primary hippocampal neurons were fully differentiated and healthy. We first detected the effect of H_2_O_2_ on the cell viability of primary hippocampal neurons. After exposure of primary hippocampal neurons to H_2_O_2_ for 24 h, MTT assay showed that H_2_O_2_ caused a concentration-dependent reduction of cell viability. After stimulation with 200 μmol/L H_2_O_2_ for 24 h, the cell viability had dropped to ~60% (*n* = 6), (*p* < 0.05, Figure 1b). Therefore, we selected the concentration of 200 μmol/L of H_2_O_2_ to stimulate the neurons for 24 h in subsequent experiments to induce oxidative stress.

### 2.2. H_2_O_2_ Induced Apoptosis and Death of Primary Cultured Hippocampal Neurons

**Figure 2:**
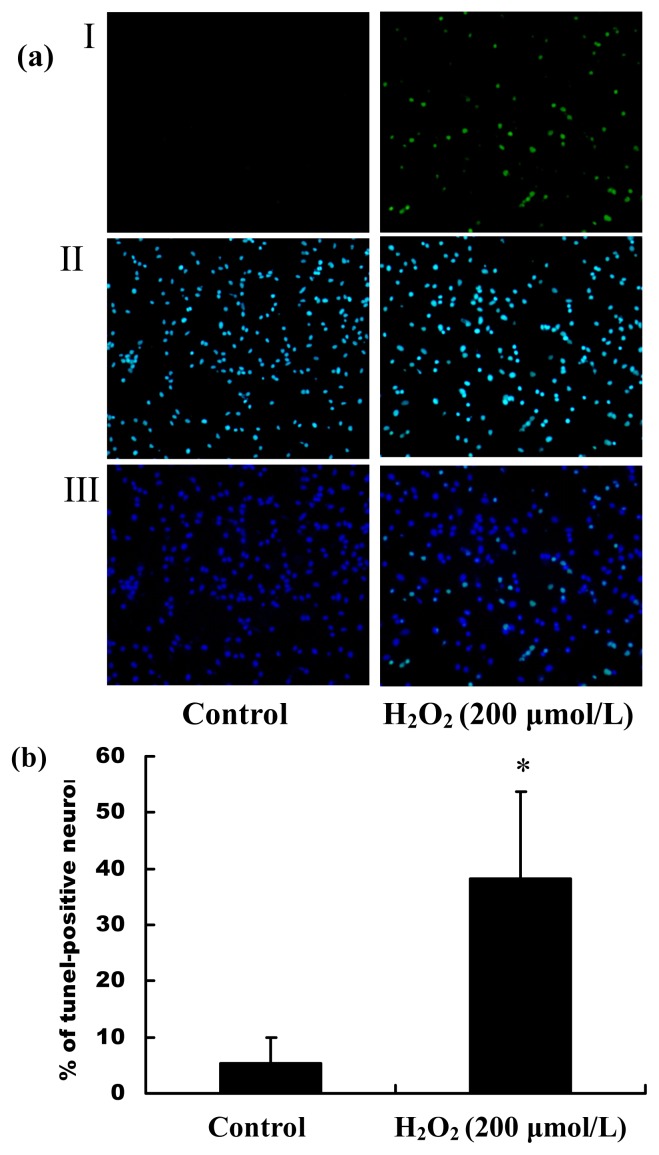
The apoptosis percentage of primary hippocampal neurons induced by H_2_O_2_. Cells were stained with TUNEL and Hoechst 33258 after stimulation with 200 μmol/L H_2_O_2_ for 24 h. (**a**) Morphological apoptosis was determined by TUNEL assay. Green-stained cells were TUNEL-positive cells (I). All nuclei were stained with Hoechst 33258 (II). The merge of I and II is III (200×); (**b**) The ratio of TUNEL-positive primary hippocampal neurons to the total cells. * *p* < 0.01 *versus* control. All data were expressed as mean ± SD of three experiments. The images shown are representative images.

**Figure 3:**
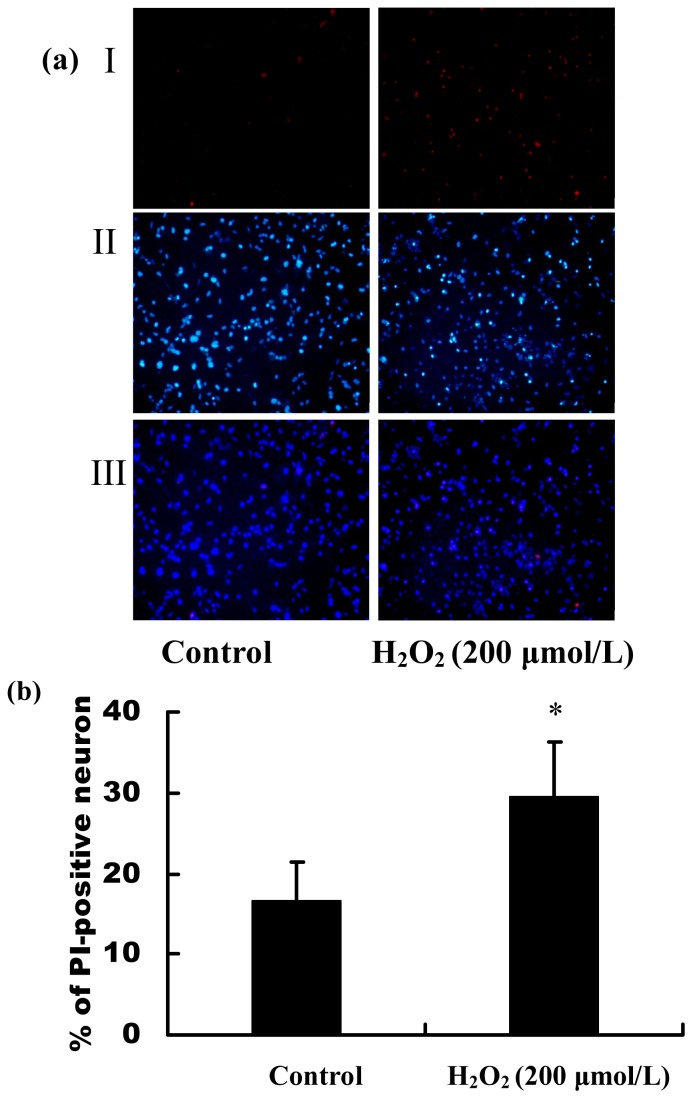
The death percentage of primary hippocampal neurons induced by H_2_O_2_. Cells were stained with PI and Hoechst 33258 after stimulation with 200 μmol/L H_2_O_2_ for 24 h. (**a**) Morphological cell death was determined by PI staining. Red stained cells were PI-positive cells (I). All nuclei were stained with Hoechst 33258 (II). The merge of I and II is III (200×); (**b**) The ratio of PI -positive primary hippocampal neurons to the total cells. * *p* < 0.01 *versus* control. The images shown are representative images.

Following our observations of the decrease in the cell viability by H_2_O_2_ stimulation, we subsequently examined whether H_2_O_2_ treatment also induced changes in apoptosis and necrosis of primary hippocampal neurons. The cell apoptosis and death rate were detected by TUNEL and PI staining, respectively. The positive stained cells were calculated by counting five randomly selected fields, and results were expressed as % positive cells/total cells ± SEM. As shown in Figure 2, the percentage of TUNEL-positive hippocampal neurons in the total cells induced by H_2_O_2_ was significantly increased, (*p* < 0.01), suggesting that H_2_O_2_ induced the primary hippocampal neuron apoptosis. The results of PI staining showed that the percentage of PI-positive hippocampal neurons in the whole cell population induced by H_2_O_2_ was significantly increased, (*p* < 0.01, Figure 3), suggesting that H_2_O_2_ induced hippocampal neuron necrosis. Taken together, the progressive reduction of cell viability caused by H_2_O_2_ was a mixture of apoptosis and necrosis.

### 2.3. Identification of Neuronal miRNAs Modulated by Oxidative Stress

To investigate whether neuronal miRNAs modulated by oxidative stress play roles in AD pathology, the expression profile of miRNAs in the primary hippocampal neurons were identified by GeneChip miRNA 2.0 Array. The result showed that there were 101 deregulated miRNAs in the primary hippocampal neurons after exposure to 200 μmol/L H_2_O_2_ for 6 h, in which 64 miRNAs were up-regulated and 37 miRNAs down-regulated. Of the 101 distinguished miRNAs, 17 miRNAs changed above 1.5-fold. Of these, 12 (mir-9, mir-200c, mir-708, mir-377, mir-26b, mir-296, mir-369, mir-32, mir-1965, mir-1190, mir-135b and mir-201) were differentially up-regulated and five (mir-291a, mir-190b, mir-297c, mir-713 and mir-470) were differentially down-regulated.

### 2.4. Deregulated miRNAs May Take Part in Regulating the Key Pathways Altered in AD

A single miRNA is predicted to regulate many target protein-coding mRNAs completely or partially at the posttranscriptional level, while a single gene can also be regulated by many miRNAs. So changes in miRNAs expression may play important roles in biological functions [18]. To identify the biological processes most relevant to the deregulated miRNAs by H_2_O_2_, we performed enrichment analysis on predicted target genes. TargetScan Mouse v5.2 and DAIAN were used to generate lists of target genes regulated by miRNAs changed upon 1.5-fold. Then, the lists were sent to the bioinformatic database DAVID. The results of pathway enrichment analysis provided by Kyoto Encyclopedia of Genes and Genomes (KEGG) showed that these miRNAs may be involved in the regulation of cell growth, differentiation, apoptosis, neurotrophin transduction, signal transmission, cancer development and so on. Mitogen-activated protein kinase (MAPK) signaling pathway was one of the most significant pathways to be affected by 74 target genes of miR-708, miR-296, miR-200c, miR-377 and miR-1190 (Table 1). These five miRNAs were all up-regulated after H_2_O_2_ stimulation. Furthermore, the Neurotrophin signaling pathway, Axon guidance, Steroid biosynthesis and Insulin signaling pathways were affected by miRNAs predicted target genes.

### 2.5. Validation of Deregulated miRNAs

**Figure 4:**
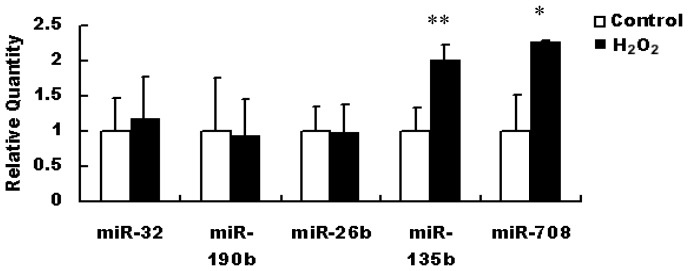
Real-time quantification of chosen miRNAs in primary hippocampal neurons exposure to H_2_O_2_ (200 μmol/L) for 6 h. *T*-test *p*-value significance: ** *p* < 0.01, * *p* < 0.05. Expression was normalized to snoRNA U6. Three replicates of each qRT-PCR were performed.

Quantitative real-time PCR was used to validate the microarray results. Due to low amounts of RNA obtained from primary hippocampal neurons, we chose five significantly deregulated miRNAs to perform quantitative real-time PCR experiments. This selection was based on the relevance of the five miRNAs to AD pathology, their expression levels and the results of bioinformatic analysis. It was showed that miR-135b (*p* < 0.01) and miR-708 (*p* < 0.05) expressed at higher levels in H_2_O_2_-treated primary hippocampal neurons compared with control cells, consistent with the results of microarray (Figure 4).

### 2.6. Functional Analysis of Targets of miR-135b and miR-708

To further illuminate the possible roles of miR-135b and miR-708 in the pathogenesis of AD, Gene Ontology (GO) enrichment for biological processes and molecular functions of miR-135 and miR-708 targets were performed. The results revealed that miR-135 was related to DNA recombination, protein ubiquitination, protein amino acid autophosphorylation and transcription factor binding (Table 2). Analysis of miR-708 targets showed that miR-708 mainly took part in the positive regulation of small GTPase-mediated signal transduction, protein amino acid phosphorylation, positive regulation of cell proliferation, neuron development, *etc.* (Table 3). Taken together, deregulated miRNAs induced by oxidative stress may play important roles in the pathogenesis of AD by influencing protein ubiquitination and phosphorylation through MAPK signaling pathway.

## 3. Discussion

Oxidative stress has a major role in many pathological conditions, such as neurodegenerative disorders, cardiovascular diseases, diabetic vasculopathy and atherosclerosis [19]. ROS, which include H_2_O_2_, superoxide anion and hydroxyl radicals, have been demonstrated to inhibit cell growth and to induce cell death and senescence. In the current study, the cultured primary hippocampal neurons *in vitro* were immunostained with the synaptic marker, MAP-2, and were shown to be fully differentiated and healthy, which ensured the accuracy of the subsequent results. Then, we developed a cell model of oxidative stress by stimulating the primary hippocampal neurons with different concentrations of H_2_O_2_. The results of MTT assay showed that H_2_O_2_ caused a dramatic reduction of cell viability in concentration-dependent manner. The measurements of neuron apoptosis and cell death by TUNEL and PI staining showed H_2_O_2_ induced the primary hippocampal neuron apoptosis and necrosis, suggesting that the mixture of cell apoptosis and necrosis contributed to the progressive reduction of cell viability caused by H_2_O_2_. MicroRNAs (miRNAs), which regulate the stability and/or the translational efficiency of target messenger RNAs (mRNAs), have been ascribed diverse functions, including regulation of proliferation, differentiation, senescence and death [20].

Recently, the major research advances pertaining to the possible role and function of miRNAs in neurodegenerative diseases, such as AD, has provided novel perspectives to the pathogenesis of increasingly prevalent, age-related diseases in human. An important role of miRNAs has been shown in a large number of specific neurological processes, including synaptic plasticity, neuroplasticity and stress responses [21,22]. The profiles of miRNAs have shown to be altered in several regions of AD brains [23,24]. And, their dysfunction may have direct relevance to the pathogenesis of AD. However, the oxidative stress-effect on miRNAs expression profiles in hippocampal neurons has not been investigated, and little is known on the potential role of ROS-modulated miRNAs on cell function, which may be an important contributor to the etiology and progression of age-related diseases. In the present study, the result of microarray analysis showed that H_2_O_2_ mediated a total of 101 deregulated miRNAs in the primary hippocampal neurons, in which 64 miRNAs were up-regulated and 37 miRNAs were down-regulated. Of the 101 distinguished miRNAs, 17 miRNAs changed above 1.5-fold. We have identified that H_2_O_2_ changed the expression profiles of miRNAs that was not only remarkably deregulated, but occurred rapidly, with six hours of H_2_O_2_ treatment, consistent with others reported [25]. These deregulated miRNAs potentially had an important effect in biological pathways essential for special brain function relevant to AD [24].

It was considered that miRNAs conducted themselves as key regulators in maintaining transcriptome homeostasis [26]. To analyze the possible biological effects of deregulated miRNAs after H_2_O_2_ stimulation, we predicted the target mRNAs of changed miRNAs and performed an enrichment analysis of multiple miRNA target genes by KEGG. The results showed that H_2_O_2_-induced miRNAs mainly took part in the MAPK pathway, Neurotrophin signaling pathway, Axon guidance, Steroid biosynthesis and Insulin signaling pathway. Among them, the MAPK pathway was one of the most significant candidate pathways to be affected by H_2_O_2_-induced up-regulated miRNAs. The up-regulated miRNAs, including miR-708, miR-296, miR-200c, miR-377 and miR-1190, were all strongly predicted to affect target genes involved in the MAPK pathway. This signaling cascade is involved in a number of cell functions, including cell development, differentiation, synaptic plasticity and learning. In recent years, it has been reported that MAPK is essential for LTP formation in the rodent hippocampus [27]. Thus, we hypothesized that H_2_O_2_-induced miRNAs deregulation of MAPK cascade may partly contribute to the loss of synaptic function in AD.

Based on the relevance to neurodegenerative disorders, their expression levels (total counts in hippocampus) and the possible functions predicted by KEGG considered [25], we chose five miRNAs, including miR-32, miR-196b, miR-26b, miR-708 and miR-135b, to validate microarray results among 17 miRNAs. Among them, miR-135b and miR-708 displayed a significant up-regulation in primary hippocampal neurons with H_2_O_2_ stimulation for 6 h. The regulation of miRNA on its target genes is a rapid process, and the oxidative stress plays an important role in the onset of neurodegenerative disorders. In this study, we mainly focused on the levels of miRNAs in the early period of oxidative stress. Therefore, after exposure with H_2_O_2_ for 6 h, quantitative real-time PCR was used to investigate the levels of miRNAs. The aim of other experiments was to demonstrate that we could develop an oxidative stress cell model successfully using H_2_O_2_ stimulation. In addition, because the microarray analysis was just for roughly screening the altered miRNA, but the quantitative real-time PCR was for measuring the level of miRNA expression precisely, we think the discrepancy between quantitative real-time PCR and microarray analysis was possible and common.

The miRNA participated pathway analysis involving miR-708 has been previously studied in other reports about lung cancer [28], childhood acute lymphoblastic leukemia [29] and renal cancer [30]. The Enrichment for Gene Ontology (GO) term of miR-708 targets revealed that miR-708 mainly took part in the process of cell apoptosis, which might explain the results of TUNEL and PI staining. Bioinformatic analysis of miR-708 target genes showed that five genes (Map3k13, Kras, Rap1b, Nras and Csf1) were predicted to affect the MAPK signaling pathway. Among them, K-ras and N-ras were also participated in the Neurotrophin signaling pathway and Axon guidance, which play important roles in neuronal functions. Given that miR-708 is upregulated in mouse hippocampal neurons induced by oxidative stress, it is, therefore, likely that aberrant expression of miR-708, at least in part, contributes to the pathology of AD.

It is reported that miR-135b, one important member of the miR-135 family, played essential roles in development of many tumors, such as anaplastic large cell lymphoma [31], osteosarcoma [32] and colon cancer [33]. We found that the level of miR-135b was significantly upregulated in the primary hippocampal neurons by H_2_O_2_ treatment. Recently, one study confirmed that Siah, a predicted target of the miR-135 family, regulated neuronal migration, development of nervous system and morphogenesis of the brain by inducing Pard3A protein ubiquitination [34]. In addition, PRP19beta, another miR-135 target gene, was founded to inhibit neurons differentiation and stimulate glial cells growth [35]. Taken together, it can be predicted that miR-135b-participated hippocampal neuron impairment is regulated, at least in part, via decreasing hippocampal Siah and PRP19beta levels.

## 4. Experimental Section

### 4.1. Materials

Healthy senescence accelerated mouse-resistant/1 (SAMR1) was obtained from the Animal Center of Beijing University Medical Department. Dulbecco’s Modified Eagle’s Medium (DMEM), fetal bovine serum (FBS), neurobasal medium and B27 were purchased from Invitrogen. Hydrogen peroxide (H_2_O_2_), trypsin, poly-l-lysine, Hoechst33258, Propidium iodide (PI), 3-(4,5-dimethylthiazol-2-yl)-2 and 5-diphenyltetrazolium bromide (MTT) were provided by Sigma-aldrich (St. Louis, MO, USA). The Dead End™ Fluorometric TUNEL System was purchased from Promega. The MAP-2 (w14) pAb Reagent was purchased from Bioword technology.

### 4.2. Cell Culture

Primary hippocampal neuron cultures were obtained from embryonic day-18 or day-19 (E18 or E19) hippocampi of SAMR1 mice. Briefly, the hippocampi, freed of meninges and vessels, were mechanically dissociated with microforceps. After treatment with 0.125% trypsin for 15 min at 37 °C in phosphate balanced salt (PBS) solution, the hippocampal cells were washed in DMEM with 20% FBS to stop trypsin activity. Then, the cells were resuspended in DMEM supplemented with 20% FBS and seeded onto 0.1 g/L poly-l-lysine-coated plates. The cultures were incubated in a humidified atmosphere of 95% air and 5% CO_2_. After cells attached to the substrate, the culture medium was changed to neurobasal medium supplemented with 2% B27, 100 kU/L of penicillin and streptomycin, followed by incubation for seven days with half of the neurobasal medium being changed every three days to ensure cell maturation.

### 4.3. Immunocytochemistry

Dishes containing mouse primary grown for 7 DIV (days *in vitro*) were fixed with 4% paraformaldehyde. Cells were permeabilized with 0.3% Triton and goat serum in PBS and then stained with primary antibodies to rabbit MAP-2 protein (1:200, Bioworld, Louis Park, MN, USA). Antibody staining was visualized using Fluorescein-5-isothiocyanate (FITC)-labeled secondary antibodies (1:100, CWBio, Beijing, China). All cell nuclei were stained with Hoechst 33258. Pictures were taken with a fluorescence microscope (Nikon, Tokyo, Japan). Purity of neuronal populations in cultures was assessed by the ratio of MAP2-positive cells to total cells.

### 4.4. Cell Viability

Primary cultured hippocampal neurons were treated with 0, 100, 200, 400 and 800 μmol/L H_2_O_2_ (Sigma-aldrich, St. Louis, MO, USA) diluted with supplemented neurobasal medium at 37 °C in a humidified atmosphere of 95% air and 5% CO_2_. After 24 h, cell viability was assessed by conventional MTT assay. Briefly, MTT (5 g/L) was added to the cell medium at a final concentration of 0.5 g/L. After incubating the cells at 37 °C for 4 h, the MTT-containing medium was replaced with DMSO to dissolve the water-insoluble formazan salt. Then, the absorbance was measured by Multiskan Ascent at 490 nm (Thermo Scientific, Wilmington, DE, USA). The neuronal survival was expressed using OD value.

### 4.5. Measurements of Neuron Apoptosis and Cell Death Induced by H_2_O_2_

Primary culture hippocampal neurons were treated with 200 μmol/L or without H_2_O_2_ for 24 h. Apoptotic cells were measured by terminal deoxynucleotide transferase dUTP nick end labeling (TUNEL) fluorescent staining, as described previously [36]. TUNEL fluorescent staining was done according to the manufacturer’s protocol. Cell death was measured by nucleic acid labeling with PI (Propidium iodide, Sigma-aldrich, St. Louis, MO, USA). For PI staining, the cultured hippocampal neurons were incubated with 5 mg/L PI for 20 min after treatment with H_2_O_2_ (200 μmol/L) for 24 h. Then, the cell nuclei were stained with Hoechst 33258 (20 mg/L) for 5 min at room temperature. The number of TUNEL/PI-positive neurons and total cells were counted under a fluorescence microscope (Nikon, Tokyo, Japan). The data were shown as the ratio of apoptotic/dead cells to total cells.

### 4.6. Microarray Analysis of miRNA Expression

Total RNA (including miRNAs) were extracted from mouse primary hippocampal neurons using the BiooPure RNA Isolation Kit (Bioo Scientific, Austen, TX, USA) according to the manufacturer’s recommendations. The quantity of RNA was assessed on a NanoDrop 1000 spectrophotometer (Thermo Fisher Scientific, Waltham, MA, USA). Total RNA (100 ng) were individually processed for miRNA expression profiling on a GeneChip miRNA 2.0 Array (Affymetrix, San Francisco, CA, USA).

### 4.7. Bioinformatic Analysis of Deregulated miRNAs

The expression microarray of miRNAs provided a large variety of gene list involved in oxidative stress. In our study, we selected miRNAs changed upon 1.5-time for computational prediction using TargetScan web platform [37] or DAIAN [38]. For functional analysis of miRNAs targets, we used the DAVID (Database for Annotation, Visualization and Interrogated Discovery) bioinformatic resources to extract biological features associated with miRNAs target gene lists [39]. The software performs an enrichment analysis of miRNA target genes with all known GO-term analysis and KEGG pathways.

### 4.8. Quantitative Real-Time PCR

Briefly, RNAs from primary cultured hippocampal cells were isolated with a BiooPure RNA Isolation Kit (Bioo Scientific, Austen, TX, USA). cDNA synthesis was performed using 300 ng small RNA, M-MuLV reverse transcriptase (Fermentas, Burlington, ON, Canada) and miRNA RT primer (Ribo Bio, Guangzhou, China). The reaction was performed at 60 °C for 1 h and heated at 70 °C for 5 min; 1 μL template from each RT reaction mixture and 0.5 μmol/L miRNA PCR primers (Ribo Bio, Guangzhou, China) were used for PCR amplification. Real-time PCR detection of miRNAs was carried out using SYBR-Green PCR Kit (CWBio, Beijing, China) according to the manufacturer’s instructions, and miRNAs were normalized to the level of mouse U6 snRNA. The PCR was performed with the following cycle: 95 °C, 10 s and 95 °C, 5 s; 60 °C, 1 min, 30 cycles.

### 4.9. Statistical Analysis

All data were presented as mean ± SD. Statistical analysis was determined by the independent Student *t* test and one-way ANOVA using SPSS 16.0 software [40]. A *p*-value cut-off 0.05 was considered statistically significant.

## 5. Conclusions

In this study, the results suggested that oxidative stress alters the miRNA expression profile of hippocampal neurons, and the deregulated miRNAs might play a potential role in the pathogenesis of neurodegenerative diseases, such as AD. Bioinformatic analysis of the deregulated miRNAs mediated by oxidative stress provides us the clues in future research to broaden our understanding of the pathogenesis of AD.

## Figures and Tables

**Table 1:** The main function predicted by bioinformatics analysis of deregulated microRNAs in mouse primary hippocampal neurons after H_2_O_2_ stimulation.

miRNA	Total targets in mouse	KEGG pathway	Targets in pathway	*p*-Value	Total counts in hippo-campus [18]
miR-135b	979	Steroid biosynthesis	4	3.05 × 10^−2^	4299
miR-708	109	Neurotrophin signaling pathway	6	2.48 × 10^−4^	9638
		MAPK signaling pathway	7	9.66 × 10^−4^	
		Axon guidance	5	2.63 × 10^−3^	
		ErbB signaling pathway	4	6.80 × 10^−3^	
		Chemokine signaling pathway	5	8.47 × 10^−3^	
		T cell receptor signaling pathway	4	1.56 × 10^−2^	
		Insulin signaling pathway	4	2.36 × 10^−2^	
miR-296	777	MAPK signaling pathway	23	1.57 × 10^−4^	163
		Hedgehog signaling pathway	6	4.18 × 10^−2^	
		Complement and coagulation cascades	7	4.96 × 10^−2^	
mir-26b	948	Regulation of autophagy	5	4.86 × 10^−2^	227704
miR-201	830	Calcium signaling pathway	13	4.79 × 10^−2^	4
		Steroid biosynthesis	4	2.28 × 10^−2^	
miR-9	888	Glycosylphosphatidylinositol (GPI)-anchor biosynthesis	6	2.67 × 10^−3^	2298920
		ECM-receptor interaction	8	4.69 × 10^−2^	
miR-200c	770	Neurotrophin signaling pathway	24	9.97 × 10^−11^	178
		Insulin signaling pathway	19	1.46 × 10^−6^	
		Axon guidance	17	1.35 × 10^−5^	
		MAPK signaling pathway	25	1.88 × 10^−5^	
		ErbB signaling pathway	13	5.00 × 10^−5^	
		mTOR signaling pathway	10	1.01 × 10^−4^	
		Apoptosis	10	3.53 × 10^−3^	
		Wnt signaling pathway	11	3.88 × 10^−2^	
miR-377	327	MAPK signaling pathway	10	1.59 × 10^−2^	1775
		Ubiquitin mediated proteolysis	7	1.50 × 10^−2^	
		TGF-beta signaling pathway	5	3.95 × 10^−2^	
		mTOR signaling pathway	4	4.53 × 10^−2^	
miR-713	810	Spliceosome	10	3.82 × 10^−2^	~
miR-470	739	ECM-receptor interaction	7	4.94 × 10^−2^	4
miR-190b	805	Valine, leucine and isoleucine degradation	6	2.68 × 10^−2^	4958
miR-1190	215	MAPK signaling pathway	9	2.10 × 10^−3^	~
		VEGF signaling pathway	4	2.95 × 10^−2^	
		Regulation of actin cytoskeleton	6	4.24 × 10^−2^	
miR-32	949	*p-value* cutoff not met	~	~	5042
miR-297	895	No Pathways predicted	~	~	13
miR-1956	3	No Pathways predicted	~	~	4
miR-369	~	~	~	~	3556
miR-291a	~	~	~	~	~

**Table 2:** Functional analysis of miR-135 targets.

	miR-135 targets GO-term Analysis	*p*-Value
**Biological process**		
1	GO:0008544~epidermis development	1.96 × 10^−3^
2	GO:0006310~DNA recombination	3.62 × 10^−3^
3	GO:0016567~protein ubiquitination	1.87 × 10^−2^
4	GO:0046777~protein amino acid autophosphorylation	1.87×10^−2^
5	GO:0002520~immune system development	2.09×10^−2^
**Cellular component**		
1	GO:0005829~cytosol	3.48 × 10^−3^
2	GO:0045177~apical part of cell	5.01 × 10^−3^
3	GO:0016324~apical plasma membrane	9.00 × 10^−3^
4	GO:0005681~spliceosome	3.74 × 10^−2^
5	GO:0044429~mitochondrial part	3.77 × 10^−2^
**Molecular function**		
1	GO:0008134~transcription factor binding	8.07 × 10^−3^
2	GO:0004713~protein tyrosine kinase activity	1.28 × 10^−2^
3	GO:0043169~cation binding	1.42 × 10^−2^
4	GO:0043167~ion binding	1.52 × 10^−2^
5	GO:0046872~metal ion binding	1.60 × 10^−2^

**Table 3:** Functional analysis of miR-708 targets.

	miR-708 targets GO-term Analysis	*p*-Value
**Biological process**		
1	GO:0051057~positive regulation of small GTPase mediated signal transduction	3.42 × 10^−3^
2	GO:0006468~protein amino acid phosphorylation	4.21 × 10^−3^
3	GO:0045444~fat cell differentiation	5.53 × 10^−3^
4	GO:0008284~positive regulation of cell proliferation	6.52 × 10^−3^
5	GO:0048666~neuron development	7.44 × 10^−3^
**Cellular component**		
1	GO:0042995~cell projection	6.54 × 10^−3^
2	GO:0030425~dendrite	1.65 × 10^−2^
3	GO:0043005~neuron projection	3.14 × 10^−2^
4	GO:0009898~internal side of plasma membrane	5.12 × 10^−2^
5	GO:0043228~non-membrane-bounded organelle	7.54 × 10^−2^
**Molecular function**		
1	GO:0004674~protein serine/threonine kinase activity	1.93 × 10^−3^
2	GO:0017076~purine nucleotide binding	4.22 × 10^−3^
3	GO:0032555~purine ribonucleotide binding	6.28 × 10^−3^
4	GO:0032553~ribonucleotide binding	6.28 × 10^−3^
5	GO:0000166~nucleotide binding	1.04 × 10^−2^
